# Clinical impact of serum survivin positivity and tissue expression of EBV-encoded RNA in diffuse large B-cell lymphoma patients treated with rituximab–CHOP

**DOI:** 10.18632/oncotarget.14636

**Published:** 2017-01-13

**Authors:** Jung Yong Hong, Kyung Ju Ryu, Chaehwa Park, Mineui Hong, Young Hyeh Ko, Won Seog Kim, Seok Jin Kim

**Affiliations:** ^1^ Department of Oncology, Asan Medical Center, University of Ulsan College of Medicine, Seoul, South Korea; ^2^ Department of Health Sciences and Technology, Samsung Advanced Institute for Health Sciences and Technology, Sungkyunkwan University, Seoul, Korea; ^3^ Samsung Biomedical Research Institute, Samsung Medical Center, Sungkyunkwan University School of Medicine, Seoul, Korea; ^4^ Department of Pathology, Kangnam Sacred Heart Hospital, Hallym University Medical Center, Seoul, Korea; ^5^ Department of Pathology, Samsung Medical Center, Sungkyunkwan University School of Medicine, Seoul, Korea; ^6^ Division of Hematology-Oncology, Department of Medicine, Samsung Medical Center, Sungkyunkwan University School of Medicine, Seoul, Korea

**Keywords:** survivin, epstein-barr virus, diffuse large B-cell lymphoma

## Abstract

Survivin is an inhibitor of apoptosis and is upregulated by Epstein–Barr virus (EBV) latent genes. Given the frequent association of EBV with lymphoid malignancies, survivin is expected to have prognostic value in diffuse large B-cell lymphoma (DLBCL). Thus, we measured the pretreatment serum level of survivin in DLBCL patients and analyzed its association with survival outcome and EBV status, as represented by EBV-encoded RNA (EBER) in DLBCL. Pretreatment serum survivin level was measured in patients registered in a prospective cohort study (*n* = 210), and serum survivin-positivity was defined as any detectable level of survivin. EBV status was determined using EBER *in situ* hybridization, and EBER-positivity was defined as 20% of examined cells showing nuclear positivity. Mean serum survivin level was higher in patients with relapsed or refractory disease than with responsive disease (59.89 pg/mL versus 17.34 pg/mL, *P* = 0.041). Serum survivin-positive patients had worse overall and progression-free survival (*P* = 0.023 and 0.022, respectively). Serum survivin positivity was associated with unfavorable characteristics including stage. In patients with non-germinal center B-cell type DLBCL, serum survivin-positive patients also had significantly worse survival than serum survivin-negative patients (*P* < 0.001). EBER-positivity was found in 6.7% (14/210) of patients, and EBER-positive patients had worse survival (*P* < 0.05). Patients having concomitant positivity for serum survivin and EBER expression (2.8%, 6/210) showed extremely poor prognosis. In the present era of rituximab in DLBCL, DLBCL with serum survivin positivity showed adverse clinical features and followed worse clinical course, especially in non-GCB subtype DLBCL. EBER-positivity was still associated with worse outcomes in DLBCL.

## INTRODUCTION

Treatment outcomes for diffuse large B-cell lymphoma (DLBCL) have been improved with the addition of rituximab to cyclophosphamide, doxorubicin, vincristine, and prednisolone (R-CHOP) chemotherapy [1]. However, up to one-third of patients still develop relapsed or refractory disease after treatment and die because of disease progression [2]. DLBCL is a widely heterogeneous disease with diverse clinical courses and a variety of molecular aberrations [3]. DLBCL subtypes at higher risk of relapsed or refractory disease include activated B-cell (ABC) subtype DLBCL and DLBCL harboring MYC rearrangement [4, 5]. Epstein–Barr virus (EBV)-positive DLBCL of the elderly has been consistently shown to have poor treatment outcomes in Asian countries [6–8]. Of note, EBV-positive DLBCL of the elderly is more common in Asian populations and is characterized by a higher frequency of the ABC-like immunophenotype and increased activity of the NF-kB and JAK–STAT pathways [7, 9]. In these DLBCL subtypes with a higher risk of relapsed or refractory disease, novel prognostic markers and treatment approaches are expected.

Survivin is a unique member of the inhibitor of apoptosis family and is one of the transcriptional targets of STAT3 and p53 proteins [10–13]. Survivin is overexpressed in diverse cancers such as non-small cell lung cancers, colorectal cancers, and lymphomas [14–17]. Previous lymphoma studies have reported that survivin overexpression, as detected by immunohistochemistry, is associated with poor clinical outcomes of DLBCL, especially ABC subtype DLBCL [17–19]. Interestingly, in terms of the diverse mechanisms of survivin upregulation, preclinical data have shown that survivin can be upregulated by EBV latent genes and is considered to contribute chemoresistance and poor clinical outcomes in EBV-associated malignancies such as EBV-positive gastric cancer and EBV-positive DLBCL [20, 21].

Despite the use of immunohistochemistry in previous studies, survivin protein concentration has not been investigated as a prognostic marker of DLBCL. Therefore, we measured the level of survivin using easily available pretreatment serum samples from DLBCL patients and analyzed the clinical impact of serum survivin level and its possible association with EBV status in DLBCL patients.

## RESULTS

### Characteristics of patients

Survivin was detected (range 1.7–2795.8 pg/mL) in the serum of 26 patients of the 210 patients (12.4%, 26/210). Receiver-operating characteristic (ROC) curve analysis could not find the optimal cut-off value of serum survivin due to low sensitivity and specificity. Patients were dichotomized into survivin-positive and -negative groups according to the presence of survivin in serum. The comparison of patients’ characteristics at diagnosis showed that serum survivin positivity was closely associated with poor performance status (ECOG 2–4, *P* < 0.001), more advanced stage (stage III–IV, *P* < 0.001), two or more sites of extranodal involvement (*P* < 0.001), bone marrow involvement (*P* < 0.001), and higher International Prognostic Index (IPI) risk groups (high–intermediate/high, *P* < 0.001). EBER expression was observed in only 14 patients, and the frequency of EBER positivity was significantly higher in serum survivin-positive patients (19.2%, 5/26) than in serum survivin-negative patients (4.9%, 9/184) (*P* = 0.018). Among the 198 patients whose immunophenotype was available, 91 (46.0%) and 107 (54.0%) were classified with the GCB and non-GCB histologic subtypes, respectively. However, the cell of origin did not differ significantly according to serum survivin positivity (Table 1).

**Table 1:** Baseline characteristic of patients

	All patients (*n* = 210)	Serum survivin (–) (*n* = 184)	Serum survivin (+) (*n* = 26)	*P*-value
**Age, no. (%)**				0.204
60 y or less	123 (58.6)	111 (60.3)	12 (46.2)	
Older than 60 y	87 (41.4)	73 (39.7)	14 (53.8)	
**Sex, no. (%)**				0.999
Male	128 (61.0)	112 (60.9)	16 (61.5)	
Female	82 (39.0)	72 (39.1)	10 (38.5)	
**Performance status, no. (%)**				**< 0.001**
ECOG 0–1	173 (82.4)	159 (86.4)	14 (53.8)	
ECOG 2–4	37 (17.6)	25 (13.6)	12 (46.2)	
**Ann Arbor stage, no. (%)**				**< 0.001**
Limited, I–II	103 (49.0)	102 (55.4)	1 (3.8)	
Advanced, III–IV	107 (51.0)	82 (44.6)	25 (96.2)	
**No. of ENI, no. (%)**				**< 0.001**
0 or 1	136 (64.8)	129 (70.1)	7 (26.9)	
2 or more	74 (35.2)	55 (29.9)	19 (73.1)	
**LDH no. (%)**				0.834
ULN or below	113 (53.8)	98 (53.3)	15 (57.7)	
Over ULN	97 (46.2)	86 (46.7)	11 (42.3)	
**IPI risk group, no. (%)**				**< 0.001**
Low/Low intermediate	130 (61.9)	126 (68.5)	4 (15.4)	
High intermediate/High	80 (38.1)	58 (31.5)	22 (84.6)	
**B symptom, no. (%)**				**0.003**
Negative	157 (74.8)	144 (78.3)	13 (50.0)	
Positive	53 (25.2)	40 (21.7)	13 (50.0)	
**Bone marrow involvement**				**< 0.001**
Negative	191 (91.0)	177 (96.2)	14 (53.8)	
Positive	19 (9.0)	7 (3.8)	12 (46.2)	
**Bulky disease, no. (%)**				0.701
No	193 (91.9)	168 (91.3)	25 (96.2)	
Yes	17 (8.1)	16 (8.7)	1 (3.8)	
**Response to front-lineTx**				0.543
CR or PR	181 (86.2)	157 (86.2)	24 (92.3)	
SD or PD	29 (13.8)	27 (13.8)	2 (7.7)	
**EBER status, no. (%)**				**0.018**
Negative	196 (93.3)	175 (95.1)	21 (80.8)	
Positive	14 (6.7)	9 (4.9)	5 (19.2)	
**Cell of origin, no. (%)**				0.528
GCB subtype	91 (46.0)	78 (45.1)	13 (52.0)	
Non-GCB subtype	107 (54.0)	95 (54.9)	12 (48.0)	

### Association of serum survivin positivity and EBER expression with survival outcome

**Figure 1:**
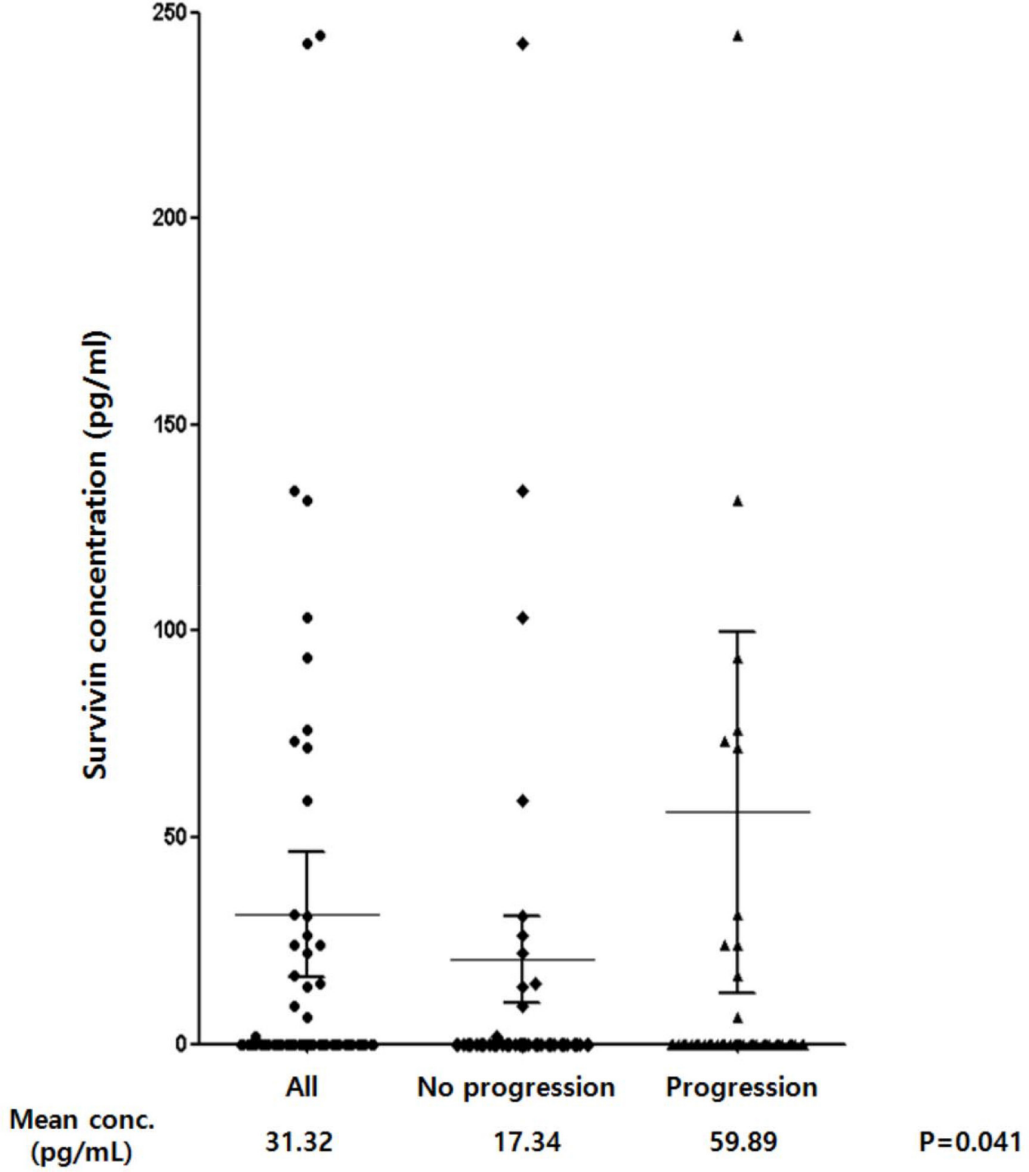
Serum levels of survivin in all patients (n = 210), patients without relapsed or refractory disease (n = 146), patients with relapsed or refractory disease (n = 64)

**Figure 2:**
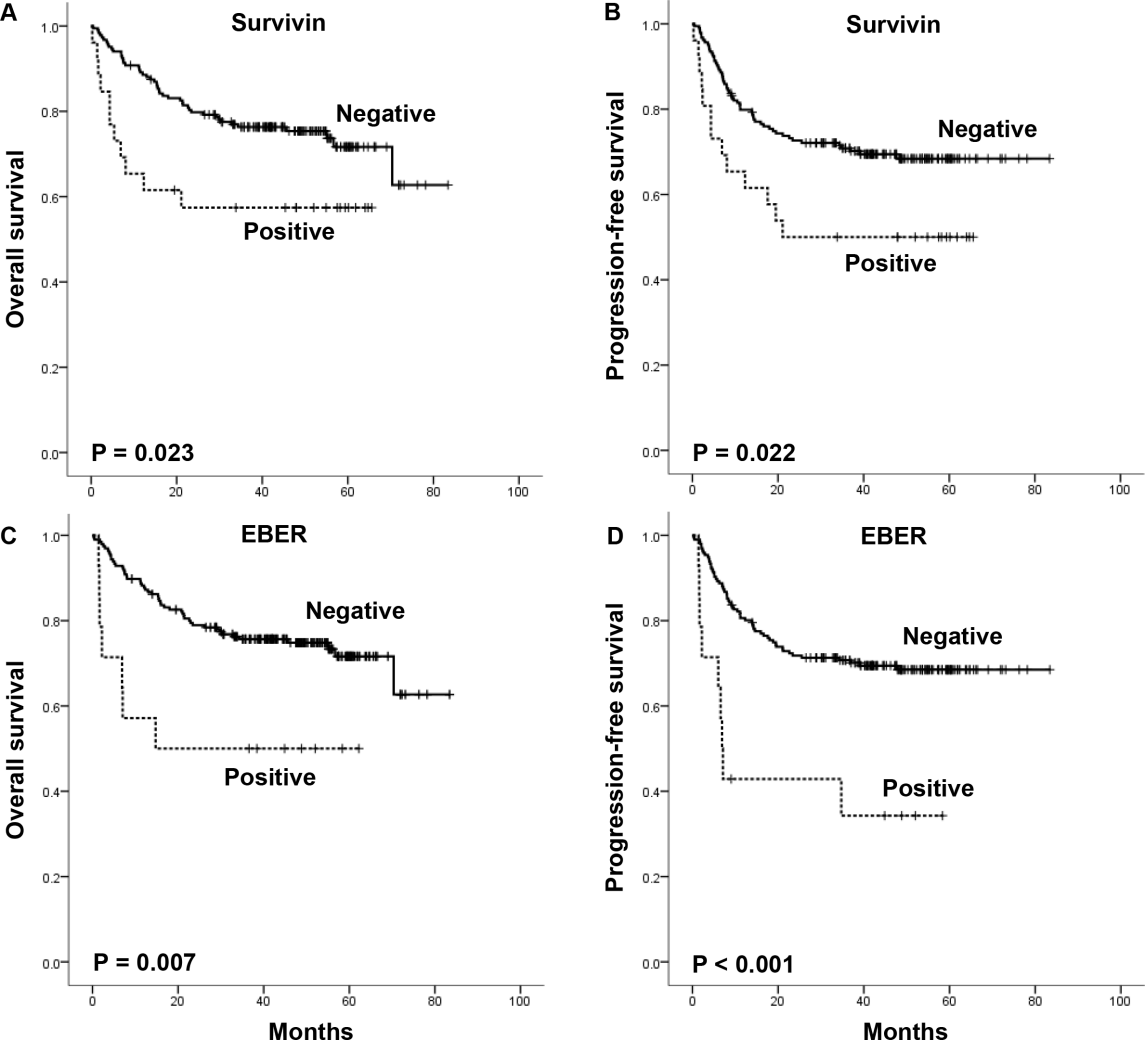
Overall survival and progression-free survival of the patients according to serum survivin positivity and EBER status

The mean serum survivin level was significantly higher in patients with, than in those without, relapsed or refractory disease (59.89 pg/mL versus 17.34 pg/mL, *P* = 0.041) (Figure 1). The percentage of serum survivin-positive patients was higher in patents with relapsed or refractory disease than in those without it showing borderline significance (18.8% versus 9.2%, *P* = 0.072). The overall 3-year OS and PFS rates of patients were 74.0% and 68.2%, respectively, for a median follow-up duration of 42.2 months (range, 0.3–83.4). Patients with serum survivin positivity showed significantly worse OS and (median OS, both not reached, *P* = 0.023) and PFS (median PFS, 21.1 months versus not reached, *P* = 0.022) compared with patients with serum survivin negativity (Figure 2A and 2B). EBER-positive patients also showed substantially worse OS and (median OS, 14.7 months versus not reached, *P* = 0.007) and PFS (median PFS, 6.9 months versus not reached, *P* < 0.001) compared with EBER-negative patients (Figure 2C and 2D).

### Prognostic value of serum survivin positivity in non-GCB and EBER-positive patients

**Figure 3:**
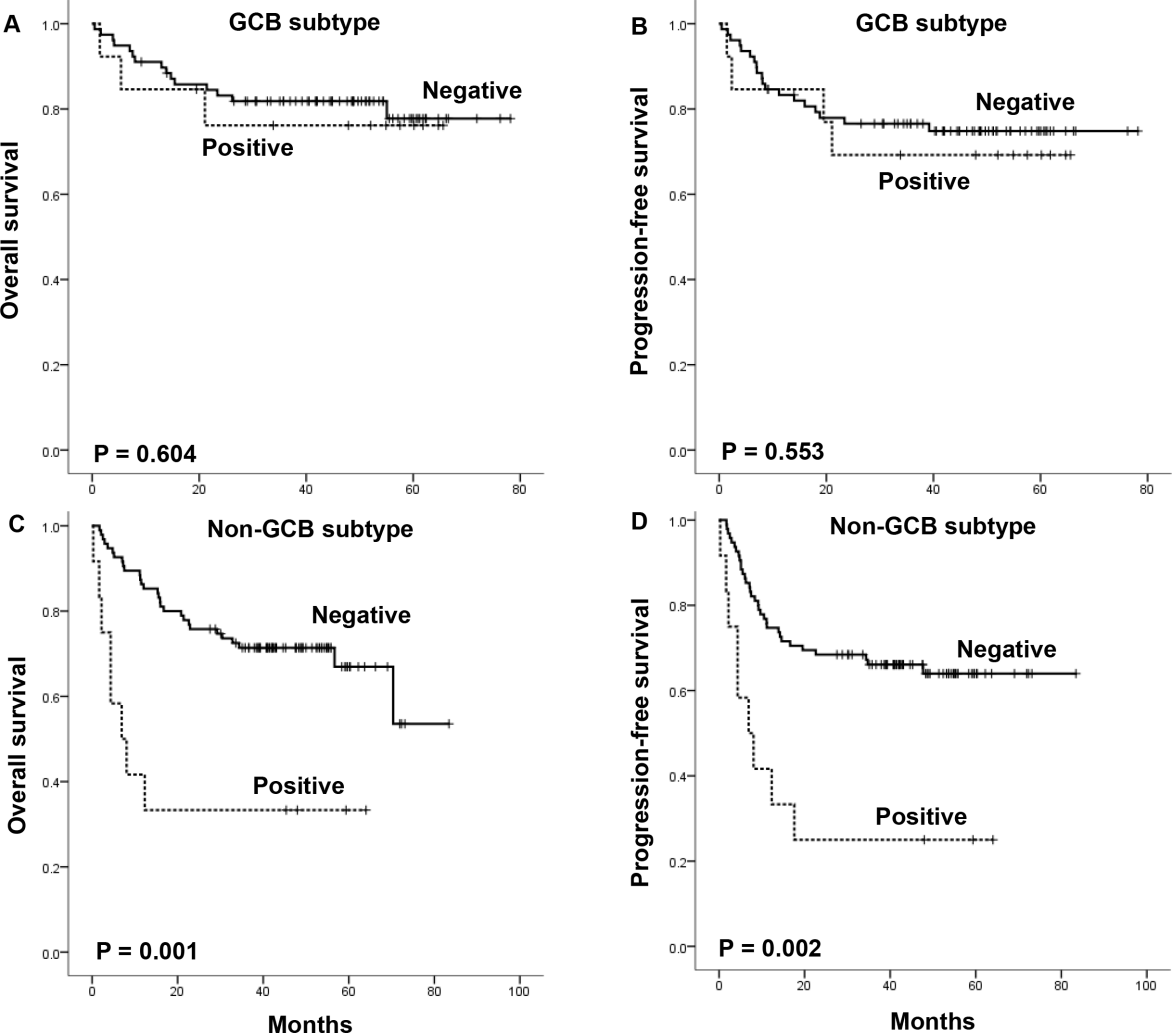
The impact of serum survivin positivity on overall survival and progression-free survival according to cell of origin of diffuse large B-cell lymphoma

**Figure 4:**
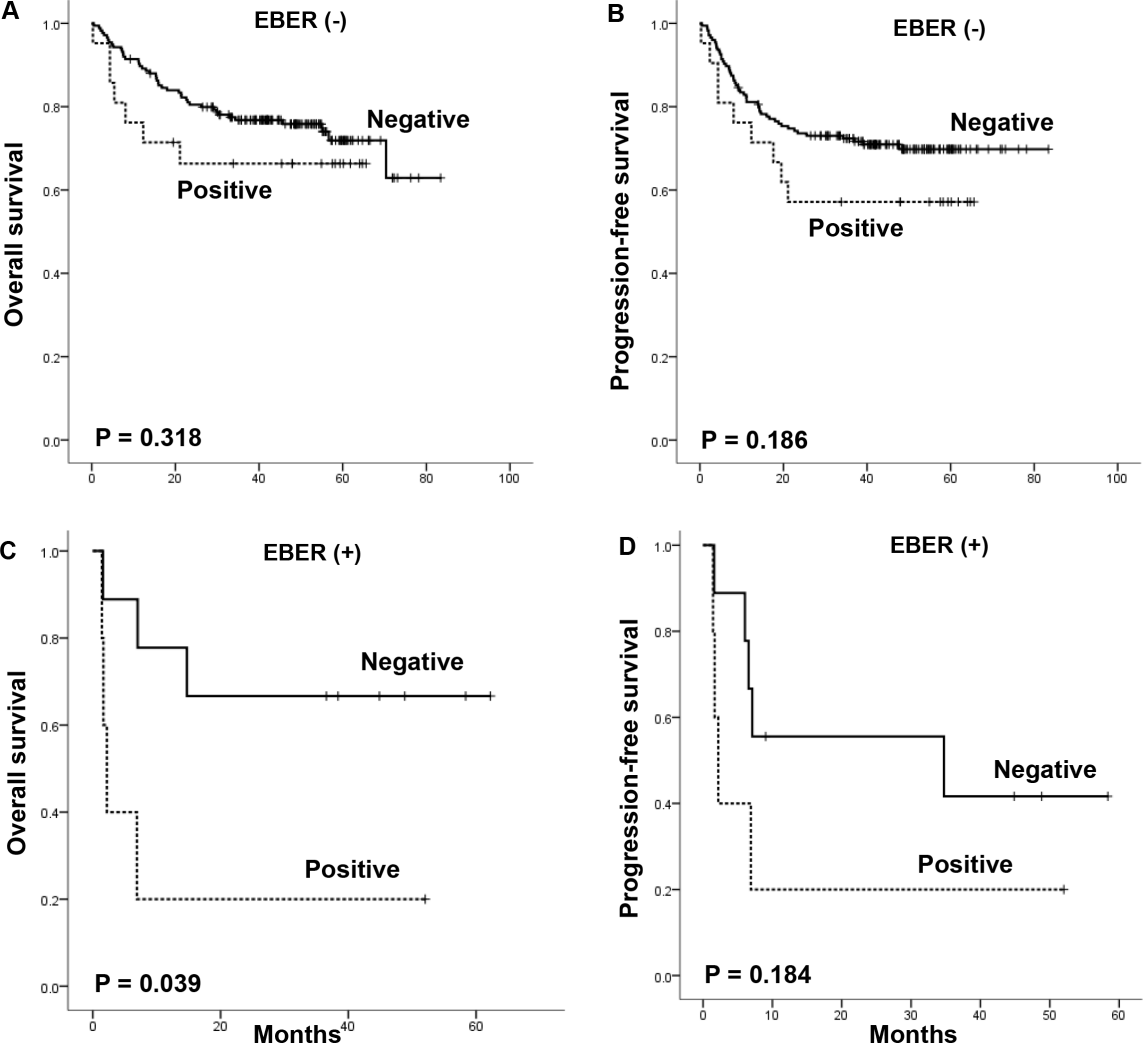
The impact of serum survivin positivity on overall survival and progression-free survival according to EBER status

We performed subgroup analysis according to the cell of origin and EBER expression status. Among 91 DLBCL patients with the GCB subtype, there were no significant differences in OS and PFS between serum survivin-positive and -negative groups (Figure 3A and 3B). However, in the 107 patients with the non-GCB subtype, serum survivin positivity was significantly associated with worse OS (median OS, 6.9 months versus not reached, *P* < 0.001) and PFS (median PFS, 6.9 months versus not reached, *P* < 0.001) compared with patients with serum survivin negativity (Figure 3C and 3D). Although the number of patients was relatively small, the association of serum survivin positivity with survival outcome was analyzed in terms of EBER expression. Serum survivin-positive patients showed a consistent trend of worse survival outcomes irrespective of EBER status (Figure 4A–4D). Of note, the subgroup with concomitant EBER positivity and serum survivin positivity (2.8%, 6/210) showed extremely poor prognosis: median OS and PFS were boths 2.2 months (Figure 4C and 4D).

### Prognostic factor analyses

In the univariate analysis, the following clinical factors were associated with worse OS: age older than 60 years (*P* < 0.001), poor performance status (ECOG 2–4, *P* < 0.001), advanced stage (stage III–IV, *P* = 0.003), two or more sites of extranodal involvement (*P* = 0.002), bone marrow involvement (*P* = 0.025), non-GCB histological subtype (*P* = 0.007), serum survivin positivity (*P* = 0.023), and EBER positivity (*P* = 0.007). Multivariate analysis showed that EBER positivity retained its significantly poor prognostic impact for worse OS [hazard ratio (HR) 2.5; 95% confidence interval (CI), 1.1–5.6; *P* = 0.025]. Other independent prognostic factors for worse OS were age older than 60 years (HR 2.4; 95% CI, 1.4–4.1; *P* = 0.001) and poor performance status (ECOG 2–4) (HR 2.8; 95% CI, 1.5–5.1; *P* = 0.001). The multivariate analyses of PFS and OS are summarized in Table 2. Serum survivin positivity lost its independent predictive power for poor prognosis in the multivariate analysis because of strong multicollinearity between survivin positivity and baseline clinical parameters, as shown in Table 1. However, in a multivariate analysis of the 107 DLBCL patients with the non-GCB subtype, serum survivin positivity showed a trend toward an association with worse OS (HR 2.3; 95% CI, 0.9–5.6; *P* = 0.067).

**Table 2:** Multivariate analysis of survival

	PFS			OS		
	HR (95% CI)		*P*	HR (95% CI)		*P*
**EBER status**			**0.001**			**0.025**
Negative	1			1		
Positive	3.4	(1.7–7.0)		2.5	(1.1–5.6)	
**Age**			**0.003**			**0.001**
60 y or less	1			1		
Older than 60 y	2.1	(1.3–3.4)		2.4	(1.4–4.1)	
**Sex**			0.500			0.925
Female	1			1		
Male	0.8	(0.5–1.4)		1.0	(0.6–1.7)	
**ECOG performance**			**0.007**			**0.001**
0–1	1			1		
2–4	2.2	(1.2–3.9)		2.8	(1.5–5.1)	
**No. of ENI**			**0.013**			0.150
0 or 1	1			1		
2 or more	2.1	(0.7–2.6)		1.6	(0.9–3.0)	
**Stage**			0.369			0.552
Limited, I–II	1			1		
Advanced, III–IV	1.3	(0.7–2.6)		1.3	(0.6–2.5)	

## DISCUSSION

Survivin is a unique member of the inhibitor of apoptosis family and plays roles in both cell survival and cell mitosis in cancer [10, 22]. Previous lymphoma studies have shown that survivin overexpression is associated with poor survival outcomes [17, 19]. However, there are some pitfalls in the interpretation of previous data. The first is the scarcity of data for uniformly R-CHOP-treated DLBCL populations. Many of the previous studies included DLBCL patients who were treated with mainly a CHOP regimen, except for the notable recent data published by Liu et al. about the International Diffuse Large B-cell Lymphoma Rituximab-CHOP Consortium Program [18]. The second pitfall is the absence of uniform criteria for defining survivin expression positivity using immunohistochemistry. Some reports used a value greater than 5% as the survivin-positive cut-off, whereas others have used cut-offs of 10%, 25%, 30%, or 45% [17–19, 23, 24]. For localizing survivin immunostaining, some reports have used cytoplasmic survivin positivity, whereas others have used nuclear positivity, mixed-type positivity, or an immunoreactivity scoring system [17–19, 23, 24]. Thus, these previous studies have reported highly variable percentages of survivin-positive DLBCL in the range of 39.3% to 84.9% [19]. Given these pitfalls, we performed this study using survivin protein concentration in easily available serum samples from DLBCL patients who were uniformly treated with R-CHOP chemotherapy.

This study revealed that serum survivin positivity was more prevalent in patients with poor performance status, more advanced stage, two or more sites of extranodal involvement, bone marrow involvement, and higher IPI risk. Liu et al. recently reported similar results that survivin expression was associated with a higher IPI risk score, higher number of extranodal disease, and higher Ki-67 index [18]. Meta-analysis also showed that positive survivin expression was associated with inferior OS and there was a significant association between survivin expression and advanced clinical stage (III and IV), higher IPI score (3–5), elevated serum LDH, presence of bone marrow involvement [19]. However, the biological mechanisms underlying release of survivin from the cell to peripheral blood are not completely understood. The distinct adverse clinical features shown in patients with serum survivin positivity suggest that serum survivin might indicate high tumor burden.

This study showed that serum survivin positivity was significantly associated with inferior survival outcomes in DLBCL patients who were uniformly treated with R-CHOP chemotherapy. Notably, subgroup analysis according to histological subtype suggested that serum survivin positivity had a more pronounced prognostic impact in patients with the non-GCB subtype DLBCL. These results are similar to those reported recently by Liu et al. [18]. Considering the unmet need for additional effective treatment for ABC subtype DLBCL, these results suggests that survivin might be a useful prognostic marker and a therapeutic target in ABC subtype DLBCL.

We also investigated possible associations between serum survivin positivity and EBER status based on preclinical evidence that survivin can be upregulated by EBV latent genes and can contribute to poor clinical outcomes of EBV-associated malignancies [21]. The percentage of EBER positive patients was significantly higher in the serum survivin-positive group than in the serum survivin-negative group (19.2% versus 4.2%). This study also reaffirmed that EBER positivity had a poor prognostic impact on DLBCL in the rituximab era. Patients with concomitant EBER positivity and serum survivin positivity showed extremely poor prognosis, with median OS and PFS both of 2.2 months.

However, this study have several limitations. To have a stronger evidence of survivin as a prognostic marker or a therapeutic target, further validation researches are needed to investigate the correlation between positive serum survivin and survivin expression in the tumor tissue. The large number of serum survivin-negative patients can skew the difference of serum survivin level between patients with or without relapsed or refractory disease. Total number of EBV-positive DLBCL patients with concurrent positive serum survivin was small. So, we should cautiously interpretate the results and the results need to be confirmed on a larger cohort. Loss of independent prognostic power of serum survivin positivity in the whole population limit the universal application of serum survivin as a prognostic marker in DLBCL. However, in the subgroup analysis of non-GCB subtype, we found a more pronounced prognostic impact of serum survivin (Figure 3) and strong trend with borderline statistical significance (HR 2.3 *P*-value = 0.067) in the multivariate analysis. This suggests serum survivin is still noteworthy for further investigation as a prognostic marker especially in non-GCB subtype DLBCL patients.

A selective survivin suppressant, YM155, demonstrates potent antitumor activities in a wide variety of cell lines and xenograft models, including lymphomas [25–27]. The first phase I clinical trial of YM155 has been published and has reported that YM155 produced an objective response in three of five patients with non-Hodgkin lymphoma [28]. In a phase II clinical trial, single-agent YM155 was well tolerated but demonstrated minimal activity in refractory DLBCL with a response rate of 2.4% [29]. However, preclinical data have demonstrated promising synergistic effects of YM155 when combined with rituximab, rituximab plus bendamustine, or a STAT3 inhibitor [30–32]. Further clinical trials using combination regimens with YM155 in the treatment of lymphomas are expected.

In summary, DLBCL patients with serum survivin positivity showed distinct adverse clinical features and followed a significantly worse clinical course, especially in those with non-GCB subtype DLBCL. In this rituximab era, EBER positivity remains a predictor of poor prognosis for patients with DLBCL. Our findings also suggest that further studies are needed to examine the feasibility of using survivin as a therapeutic target in DLBCL patients with the subtypes having higher risk of relapsed or refractory disease, such as ABC subtype DLBCL and EBV-positive DLBCL of the elderly.

## MATERIALS AND METHODS

### Patients

This study analyzed samples from DLBCL patients enrolled in our prospective cohort study between September 2008 and December 2011 (NCT#00822731). Using the following inclusion criteria, we selected 210 patients. (1) Patients should be pathologically confirmed as having DLBCL according to the World Health Organization (WHO) classification. (2) Patients should have chemotherapy-naïve, newly diagnosed DLBCL and receive R-CHOP chemotherapy as their first treatment. (3) Patients should have available pretreatment serum samples collected at diagnosis for measurement of serum survivin level. (4) Patients should have an adequate amount and quality of paraffin-embedded biopsy specimens or unstained slides for EBV-encoded RNA (EBER) *in situ* hybridization (ISH). The clinical data including disease and survival status were updated in September 2015, and the protocol was approved by the Samsung Medical Center Institutional Review Board. Written informed consent was obtained from the patients for enrollment in the prospective cohort study and the use of samples for research.

### Pathology review

The pathology of the DLBCL cases was confirmed by an expert hematopathologist (Y.H.K.) using the WHO classification. To determine the cell of origin of DLBCL, immunohistochemical staining was performed in formalin-fixed paraffin-embedded specimens using a panel of monoclonal antibodies against CD10 (Dakopatts, Copenhagen, Denmark), BCL-6 (Dakopatts), and MUM-1 (Dakopatts). Stained slides were reviewed, and the cell of origin was determined by expert hematopathologists (M.H. and Y.H.K.) according to the results of immunohistochemistry. Thus, patients were classified as having the germinal center B-cell-like (GCB) or non-GCB subtype based on the Hans algorithm, as proposed previously [33]. EBER was detected using ISH and an EBV ISH kit (Leica Microsystems, Bannockburn, IL, USA). We used EBV-negative lymphoid tissues and the hybridization mixture without EBV oligonucleotides as negative controls. A positive reaction was defined as more than 20% of examined cells showing nuclear positivity, as applied in our previous series [6, 7, 34].

### Serum survivin assay with archived serum samples

Serum survivin concentration was measured in archived frozen samples of the aforementioned prospective cohort study. Archived serum sample aliquots had been stored at –80°C and were thawed before use in the cytokine assay. The concentration of survivin, an antiapoptosis protein, was measured in serum using the Procarta cytokine profiling kit (Panomics, San Diego, CA, USA), and all measurements were performed in duplicate according to the manufacturer's instructions.

### Statistical analysis

Intergroup comparisons were performed using Fisher's exact test for categorical variables. Progression-free survival (PFS) was calculated from the date of diagnosis to the first day of disease progression, relapse, or death from any cause. Overall survival (OS) was calculated from the date of diagnosis to death. PFS and OS were censored on the last date of follow-up. Survival curves were estimated by Kaplan–Meier method, and the survival distributions were compared using the log-rank test. Multivariate analysis was performed using Cox regression analysis. *P*-values less than 0.05 were considered to be significant, and two-sided tests were used in all calculations. Statistical analyses were performed using the software package IBM PASW version 18.0 (SPSS Inc., Chicago, IL, USA).

## References

[1] Coiffier B, Lepage E, Briere J, Herbrecht R, Tilly H, Bouabdallah R, Morel P, Van Den Neste E, Salles G, Gaulard P, Reyes F, Lederlin P, Gisselbrecht C (2002). CHOP chemotherapy plus rituximab compared with CHOP alone in elderly patients with diffuse large-B-cell lymphoma. N Engl J Med.

[2] Friedberg JW (2011). Relapsed/refractory diffuse large B-cell lymphoma. Hematology Am Soc Hematol Educ Program.

[3] Alizadeh AA, Eisen MB, Davis RE, Ma C, Lossos IS, Rosenwald A, Boldrick JC, Sabet H, Tran T, Yu X, Powell JI, Yang L, Marti GE (2000). Distinct types of diffuse large B-cell lymphoma identified by gene expression profiling. Nature.

[4] Lenz G, Wright G, Dave SS, Xiao W, Powell J, Zhao H, Xu W, Tan B, Goldschmidt N, Iqbal J, Vose J, Bast M, Fu K (2008). Stromal gene signatures in large-B-cell lymphomas. N Engl J Med.

[5] Savage KJ, Johnson NA, Ben-Neriah S, Connors JM, Sehn LH, Farinha P, Horsman DE, Gascoyne RD (2009). MYC gene rearrangements are associated with a poor prognosis in diffuse large B-cell lymphoma patients treated with R-CHOP chemotherapy. Blood.

[6] Park S, Lee J, Ko YH, Han A, Jun HJ, Lee SC, Hwang IG, Park YH, Ahn JS, Jung CW, Kim K, Ahn YC, Kang WK (2007). The impact of Epstein-Barr virus status on clinical outcome in diffuse large B-cell lymphoma. Blood.

[7] Hong JY, Ko YH, Kim SJ, Kim WS (2015). Epstein-Barr virus-positive diffuse large B-cell lymphoma of the elderly: a concise review and update. Curr Opin Oncol.

[8] Sato A, Nakamura N, Kojima M, Ohmachi K, Carreras J, Kikuti YY, Numata H, Ohgiya D, Tazume K, Amaki J, Moriuchi M, Miyamoto M, Aoyama Y (2014). Clinical outcome of Epstein-Barr virus-positive diffuse large B-cell lymphoma of the elderly in the rituximab era. Cancer Sci.

[9] Ok CY, Papathomas TG, Medeiros LJ, Young KH (2013). EBV-positive diffuse large B-cell lymphoma of the elderly. Blood.

[10] Ambrosini G, Adida C, Altieri DC (1997). A novel anti-apoptosis gene, survivin, expressed in cancer and lymphoma. Nat Med.

[11] Mirza A, McGuirk M, Hockenberry TN, Wu Q, Ashar H, Black S, Wen SF, Wang L, Kirschmeier P, Bishop WR, Nielsen LL, Pickett CB, Liu S (2002). Human survivin is negatively regulated by wild-type p53 and participates in p53-dependent apoptotic pathway. Oncogene.

[12] Aoki Y, Feldman GM, Tosato G (2003). Inhibition of STAT3 signaling induces apoptosis and decreases survivin expression in primary effusion lymphoma. Blood.

[13] Gritsko T, Williams A, Turkson J, Kaneko S, Bowman T, Huang M, Nam S, Eweis I, Diaz N, Sullivan D, Yoder S, Enkemann S, Eschrich S (2006). Persistent activation of stat3 signaling induces survivin gene expression and confers resistance to apoptosis in human breast cancer cells. Clin Cancer Res.

[14] Kawasaki H, Altieri DC, Lu CD, Toyoda M, Tenjo T, Tanigawa N (1998). Inhibition of apoptosis by survivin predicts shorter survival rates in colorectal cancer. Cancer Res.

[15] Shinohara ET, Gonzalez A, Massion PP, Chen H, Li M, Freyer AS, Olson SJ, Andersen JJ, Shyr Y, Carbone DP, Johnson DH, Hallahan DE, Lu B (2005). Nuclear survivin predicts recurrence and poor survival in patients with resected nonsmall cell lung carcinoma. Cancer.

[16] Schlette EJ, Medeiros LJ, Goy A, Lai R, Rassidakis GZ (2004). Survivin expression predicts poorer prognosis in anaplastic large-cell lymphoma. J Clin Oncol.

[17] Adida C, Haioun C, Gaulard P, Lepage E, Morel P, Briere J, Dombret H, Reyes F, Diebold J, Gisselbrecht C, Salles G, Altieri DC, Molina TJ (2000). Prognostic significance of survivin expression in diffuse large B-cell lymphomas. Blood.

[18] Liu Z, Xu-Monette ZY, Cao X, Manyam GC, Wang X, Tzankov A, Xia Y, Li X, Visco C, Sun R, Zhang L, Montes-Moreno S, Dybkaer K (2015). Prognostic and biological significance of survivin expression in patients with diffuse large B-cell lymphoma treated with rituximab-CHOP therapy. Mod Pathol.

[19] Zhang Y, Wang J, Sui X, Li Y, Lu K, Fang X, Jiang Y, Wang X (2015). Prognostic and Clinicopathological Value of Survivin in Diffuse Large B-cell Lymphoma: A Meta-Analysis. Medicine (Baltimore).

[20] Lu J, Murakami M, Verma SC, Cai Q, Haldar S, Kaul R, Wasik MA, Middeldorp J, Robertson ES (2011). Epstein-Barr Virus nuclear antigen 1 (EBNA1) confers resistance to apoptosis in EBV-positive B-lymphoma cells through up-regulation of survivin. Virology.

[21] Hino R, Uozaki H, Inoue Y, Shintani Y, Ushiku T, Sakatani T, Takada K, Fukayama M (2008). Survival advantage of EBV-associated gastric carcinoma: survivin up-regulation by viral latent membrane protein 2A. Cancer Res.

[22] Mita AC, Mita MM, Nawrocki ST, Giles FJ (2008). Survivin: key regulator of mitosis and apoptosis and novel target for cancer therapeutics. Clin Cancer Res.

[23] Mitrovic Z, Ilic I, Aurer I, Kinda SB, Radman I, Dotlic S, Ajdukovic R, Labar B (2011). Prognostic significance of survivin and caspase-3 immunohistochemical expression in patients with diffuse large B-cell lymphoma treated with rituximab and CHOP. Pathol Oncol Res.

[24] Bedewy AM, Elgammal MM, Bedewy MM, El-Maghraby SM (2013). Assessing DcR3 expression in relation to survivin and other prognostic factors in B cell non-Hodgkin's lymphoma. Ann Hematol.

[25] Nakahara T, Kita A, Yamanaka K, Mori M, Amino N, Takeuchi M, Tominaga F, Kinoyama I, Matsuhisa A, Kudou M, Sasamata M (2011). Broad spectrum and potent antitumor activities of YM155, a novel small-molecule survivin suppressant, in a wide variety of human cancer cell lines and xenograft models. Cancer Sci.

[26] Kita A, Nakahara T, Yamanaka K, Nakano K, Nakata M, Mori M, Kaneko N, Koutoku H, Izumisawa N, Sasamata M (2011). Antitumor effects of YM155, a novel survivin suppressant, against human aggressive non-Hodgkin lymphoma. Leuk Res.

[27] Nakahara T, Kita A, Yamanaka K, Mori M, Amino N, Takeuchi M, Tominaga F, Hatakeyama S, Kinoyama I, Matsuhisa A, Kudoh M, Sasamata M (2007). YM155, a novel small-molecule survivin suppressant, induces regression of established human hormone-refractory prostate tumor xenografts. Cancer Res.

[28] Tolcher AW, Mita A, Lewis LD, Garrett CR, Till E, Daud AI, Patnaik A, Papadopoulos K, Takimoto C, Bartels P, Keating A, Antonia S (2008). Phase I and pharmacokinetic study of YM155, a small-molecule inhibitor of survivin. J Clin Oncol.

[29] Cheson BD, Bartlett NL, Vose JM, Lopez-Hernandez A, Seiz AL, Keating AT, Shamsili S, Papadopoulos KP (2012). A phase II study of the survivin suppressant YM155 in patients with refractory diffuse large B-cell lymphoma. Cancer.

[30] Kita A, Mitsuoka K, Kaneko N, Nakata M, Yamanaka K, Jitsuoka M, Miyoshi S, Noda A, Mori M, Nakahara T, Sasamata M (2012). Sepantronium bromide (YM155) enhances response of human B-cell non-Hodgkin lymphoma to rituximab. J Pharmacol Exp Ther.

[31] Kaneko N, Mitsuoka K, Amino N, Yamanaka K, Kita A, Mori M, Miyoshi S, Kuromitsu S (2014). Combination of YM155, a survivin suppressant, with bendamustine and rituximab: a new combination therapy to treat relapsed/refractory diffuse large B-cell lymphoma. Clin Cancer Res.

[32] Kaneko N, Kita A, Yamanaka K, Mori M (2013). Combination of YM155, a survivin suppressant with a STAT3 inhibitor: a new strategy to treat diffuse large B-cell lymphoma. Leuk Res.

[33] Hans CP, Weisenburger DD, Greiner TC, Gascoyne RD, Delabie J, Ott G, Muller-Hermelink HK, Campo E, Braziel RM, Jaffe ES, Pan Z, Farinha P, Smith LM (2004). Confirmation of the molecular classification of diffuse large B-cell lymphoma by immunohistochemistry using a tissue microarray. Blood.

[34] Hong JY, Hong ME, Choi MK, Kim YS, Chang W, Maeng CH, Park S, Lee SJ, Do IG, Jo JS, Jung SH, Kim SJ, Ko YH (2014). The impact of activated p-AKT expression on clinical outcomes in diffuse large B-cell lymphoma: a clinicopathological study of 262 cases. Ann Oncol.

